# Converting Evidence‐Based Summary of Findings Evidence Tables Into Decision Analytical, Quality Adjusted Life Years (QALY) and Life Expectancies Metrics: A Tutorial

**DOI:** 10.1111/jep.70254

**Published:** 2025-08-19

**Authors:** Iztok Hozo, Gordon Guyatt, Benjamin Djulbegovic

**Affiliations:** ^1^ Department of Mathematics Indiana University Northwest Gary Indiana USA; ^2^ Department of Health Research Methods Evidence, and Impact McMaster University Hamilton Ontario Canada; ^3^ Department of Medicine Division of Medical Hematology and Oncology Medical University of South Carolina Charleston South Carolina USA

**Keywords:** Bayesian statistics, decision analyses, decision making, evidence‐based medicine, frequentist statistics, GRADE, guidelines, inferences

## Abstract

**Rationale, Aims, and Objectives:**

We have recently succeeded in integrating evidence estimation with decision‐analytical frameworks, thereby addressing a major challenge in advancing the science of evidence‐based medicine (EBM) and clinical practice guidelines. However, the primary output of our analysis was expressed as net differences in expected utility (ΔEU) between competing treatment interventions. Although expected utility is a standard decision‐analytic metric, it is not intuitively understood by most clinicians. Here, we demonstrate how ΔEU can be converted into gains in quality‐adjusted life years (QALYs) and life expectancy (LE).

**Methods:**

We begin with GRADE (Grading of Recommendations Assessment, Development, and Evaluation) Summary of Findings (SoF) tables—the primary outputs of systematic reviews that underpin guideline recommendations—to generate ΔEU, which we subsequently convert into QALY and LE gains using the DEALE (Declining Exponential Approximation of Life Expectancy) method. We also integrate patients’ values and preferences by relating minimal important differences (MIDs)—the smallest change in an outcome that patients perceive as important enough to justify a change in management—to relative values, which reflect the preference (or weight) assigned to avoiding a specific health outcome compared to the worst outcome (mortality).

To convert a deterministic ΔEU model into a probabilistic one, we employ Monte Carlo simulation to assess the credibility of recommendations under the evidentiary uncertainty included in the SoF tables. We also provide a method to assess the impact of the certainty of evidence (CoE) on the robustness of the results.

**Results:**

We developed a user‐friendly, Excel‐based calculator for converting evidence‐based SoF tables into ΔEU, and subsequently into QALY and LE gains. We illustrate our methods by comparing the effects of short‐term versus indefinite anticoagulation for the prevention of recurrent venous thromboembolism. The complete analysis can be performed in approximately 5–10 min.

**Conclusion:**

We extend our methods to link estimation metrics commonly used in the EBM field with decision‐analytic metrics such as expected utility, QALY, and LE. We present a user‐friendly calculator that integrates all key domains underpinning contemporary guideline development.

*A conditio sine qua non* for the development of evidence‐based guidelines is the summary of the best available evidence on a given clinical topic. These summaries are typically presented in widely popular evidence and Summary of Findings (SoF) tables [1].

The SoF tables provide information about key outcomes related to the benefits and harms of competing health interventions, including details about the quantity of evidence (number of studies and participants) and the certainty (quality) of evidence (CoE), which is rated from very low to high [1, 2]. In what is referred to as “going from evidence to recommendations” [3] panel members rely on SoF tables to weigh factors such as CoE, benefits and harms, costs, equity, acceptability and feasibility (where relevant), and assessments of patients’ values and preferences (V&P). These considerations inform strong or weak (conditional) recommendations for or against a particular health intervention, following GRADE (Grading of Recommendations Assessment, Development, and Evaluation)—currently the most advanced system for developing practice guidelines [3].

The SoF inputs are based on estimation within the frequentist, with occasional use of the Bayesian framework. That is, SoF tables are typically populated with estimated mean effects of interventions, accompanied by 95% confidence intervals for all outcomes of interest. However, these estimation frameworks do not explicitly account for the *consequences*—benefits, harms, or costs—of the interventions included in the SoF tables. Integration of frequentist and Bayesian estimates is only possible through decision‐analytical frameworks that combine multiple outcomes—both benefits and harms—into a *single metric* to support decision‐making [4, 5].

We have recently succeeded in integrating evidence estimation with a decision‐analytical framework [5], thereby addressing a major challenge in advancing the science of evidence‐based medicine (EBM) and clinical practice guidelines [6]. However, the main output of our analysis was expressed as net differences in expected utility (ΔEU) between competing treatment interventions. Although expected utility is a standard decision‐analytic metric, it is not intuitively understood by most clinicians. Here, we show how ΔEU can be converted into quality‐adjusted life years (QALYs) and life expectancy (LE) gains.

## 1 Methods

As mentioned, we recently demonstrated that the guideline development process can be improved by integrating evidence estimation with decision‐analytical framework [5]. Decision analysis is the only analytical approach that combines multiple outcomes into a single metric to facilitate decision‐making. We leveraged the PICO format (Population, Intervention, Comparison(s), Outcome)—a pairwise comparison of competing management alternatives—as a conceptual basis for deriving SoF tables. We then matched these tables to simple decision‐analytical models, restricted to time frames supported by empirically verifiable evidence, to calculate which competing intervention offers the greatest benefit (net difference in expected utility; ΔEU).

The details are described in our parent paper [5], but, briefly, EU of each management strategy (Rx1 vs Rx2) is calculated as the weighted sum of mortality and morbidities (disutilities) for outcomes in the presence of the disease, for treatment1 [Rx1, D + ] vs treatment2 [or, No Treatment] [Rx2, D+]:

$$\begin{array}{l} \text{Weighted}\;\text{Disutility}\;\left(\text{WDU}\right)\left(\mathrm{Rx}1,D+\right)\\ =\text{WDU}\;\left[\mathrm{Rx}1\right]\;={\text{RV}}_{\text{mort}}\cdot \text{Mortality}\;+\;{\text{RV}}_{\text{morb}1}\cdot \text{Morbidity}1\\ \;+\;{\text{RV}}_{\text{morb}2}\cdot \text{Morbidity}2\;+\text{…}\;{\text{RV}}_{\text{morb}\_n}\cdot \text{Morbidity}\_n\;\\ \;+\;\text{…}+{\text{RV}}_{\text{adverse}\_\text{events}1}\cdot \text{AdverseEvents}1\;\\ \;+\;{\text{RV}}_{\text{adverse}\_\text{events}2}\cdot \text{AdverseEvents}2\\ \;+\;\text{…}+{\text{RV}}_{\text{adverse}\_\text{events}\_n}\cdot \text{Adverse}\_\text{events}\_n \end{array}$$

$$\begin{array}{l} \text{Weighted}\;\text{Disutility }\left(\mathrm{Rx}2,D+\right)\\ =\text{WDU}\;\left[\mathrm{Rx}2\right]={\text{RV}}_{\text{mort}}\cdot \text{Mortality}\;+\;{\text{RV}}_{\text{morb}1}\cdot \text{Morbidity}1\\ \;+\;{\text{RV}}_{\text{morb}2}\cdot \text{Morbidity}2\;+\text{…}\;{\text{RV}}_{\text{morb}\_n}\cdot \text{Morbidity}\_n\\ \;+\;\text{…}+\;{\text{RV}}_{\text{adverse}\_\text{events}1}\cdot \text{AdverseEvents}1\\ \;+\;{\text{RV}}_{\text{adverse}\_\text{events}2}\cdot \text{AdverseEvents}2\\ \;+\;\text{…}+\;{\text{RV}}_{\text{adverse}\_\text{events}\_n}\cdot \text{Adverse}\_\text{events}\_n \end{array}$$

[NB By default, we set $RV_{\mathrm{mort}}=1$, and all other relative values are compared to the mortality, hence $RV_{*}\leq RV_{\mathrm{mort}}=1$.]

Then,

EU[Rx1]=1−WDU[Rx1]

EU[Rx2]=1−WDU[Rx2]

*Differences in expected utilities (ΔEU)* are then given as

ΔEU=NetBenefits(NB)=EU[Rx1]−EU[Rx2]

The treatment with the larger net benefit (NB) is considered the better management option. However, despite its theoretical and practical advantages, the ΔEU metric is often difficult for clinicians to interpret. For example, it may be unclear what it means when indefinite antithrombotic therapy for secondary prevention in patients with unprovoked venous thromboembolism (VTE) is found to be superior to discontinuing anticoagulant treatment by ΔEU = −0.0189.

Fortunately, it is possible to convert ΔEU into more familiar metrics—quality‐adjusted life years (QALY) gains and gains in life expectancy (LEG). Many methods exists to perform these calculations [7]—some more sophisticated than others—but to meet the demand for simple and understandable methods that can be used quickly at the bedside or by busy guideline panels, we use the popular DEALE (Declining Exponential Approximation of Life Expectancy) method [8, 9]. Here, we present a new calculator for developing practice recommendations, using a straightforward application of DEALE to convert ΔEU into QALY and LEG for patients of a given age as follows:

(1) $$\text{QALY}\;\mathrm{gains}=1/\left({\text{Mort}}_{\_\text{age}\_\text{sex}\_\text{race}\;}+\text{WDU}\left[\mathrm{Rx}1\right]\right)-1/\left({\text{Mort}}_{\_\text{age}\_\text{sex}\_\text{race}\;}\;+\text{WDU}\left[\mathrm{Rx}2\right]\right)$$

(2) $$\text{LE}\;\mathrm{gains}=1/\left({\text{Mort}}_{\_\text{age}\_\text{sex}\_\text{race}\;}+{\text{RV}}_{\text{mort}}\cdot \text{Mortality}\left[\mathrm{Rx}1\right]\right)-1/\left({\text{Mort}}_{\_\text{age}\_\text{sex}\_\text{race}\;}\;+{\text{RV}}_{\text{mort}}\cdot \text{Mortality}\left[\mathrm{Rx}2\right]\right)$$

**Figure 1:**
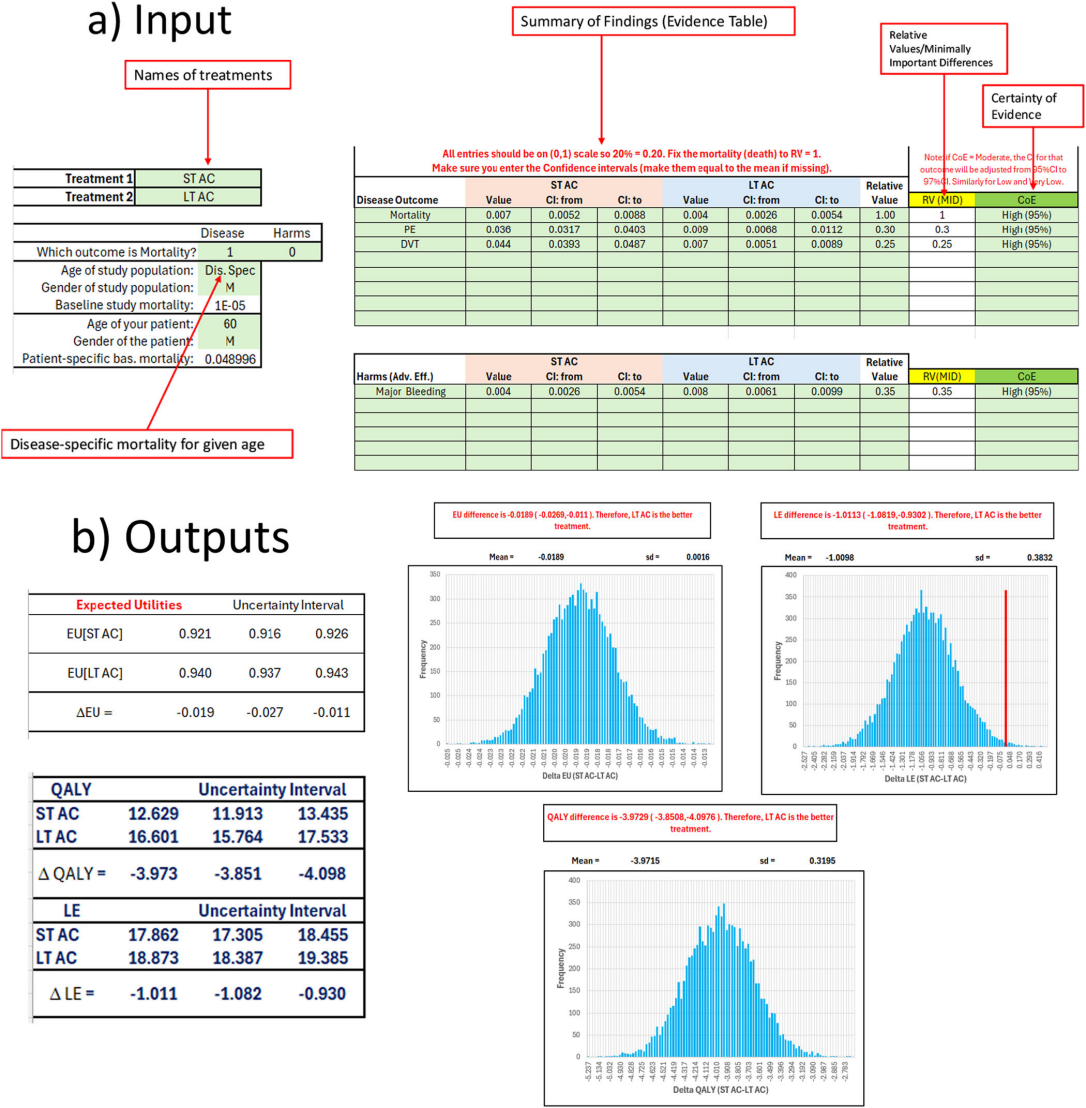
Screenshots of the calculator for determining expected utilities, QALYs (quality‐adjusted life years), and LE (life expectancy). (a) Inputs (b) Outputs. (See the main text and Excel calculator for further details).

Figure 1 shows screenshots of the Excel calculator (which can be freely downloaded from the Appendix). The calculator includes input fields (highlighted in green and yellow) and outputs displaying ΔEU, QALY gains, and LE gains of comparative treatment effects, along with their associated uncertainties.

Briefly, the following steps should be followed:

### 1.1 Input

1. Copy the names of interventions and data from the original GRADE SoF tables into the calculator's SoF table.
  Currently, the calculator allows copying up to eight disease‐related outcomes and up to six adverse events (harms). All values should be expressed in terms of absolute event rates, as this is the most effective way to communicate treatment benefits and harms [1]. Make sure that the choice of a decision model adheres to the assumptions described in our parent paper [5].
  Figure 1 shows an example of an evidence table comparing the effects of indefinite (long‐term, LT) versus discontinued (short‐term, ST) antithrombotic therapy for secondary prevention in patients with unprovoked VTE.
2. Enter data on patients' values and preferences (V&P).
  As discussed in the parent paper [5], patients’ V&P regarding the importance of avoiding one outcome over another are expressed as relative values (RV). In the calculator, this is implemented as a ratio of the weights a decision‐maker (e.g., patient) assigns to a given health outcome relative to the worst possible outcome—typically mortality, which is anchored at an RV of 1 = 100%. For example, in the figure, the RV for pulmonary embolism (PE) is assigned a weight of 0.30, meaning that avoiding death is 3.3 times more important than avoiding PE (100/30). Similarly, the RV for DVT is 0.25, and for major bleeding, 0.35 [5].
  Unfortunately, patient‐specific V&P data are often unavailable. However, physicians can elicit them at the bedside using one of many techniques described in the literature [4, 7, 10]. Guideline panel members adhering to GRADE methods are frequently asked to express the minimal important difference (MID) for each outcome [11]. The MID represents the smallest change in an outcome that patients—or guideline panels and other knowledgeable surrogates—perceive as important enough to justify a change in management, in the absence of excessive cost or harm.
  The MID can be linked to the psychological concept of “acceptable regret” [12, 13, 14, 15, 16],
  which describes situations in which a loss in utility—a small fraction of benefits or harms—resulting from a wrong decision is not particularly burdensome to the decision‐maker (patient). This occurs when the acceptable regret is smaller than the MID. MIDs capture the threshold at which changes become meaningful to patients and can be transformed into relative values expressing the comparative importance of different outcomes.
  The appropriate formula to relate MID to RV is:
(3) RV=1/(OutcomeMID÷DeathMID)
  A larger MID implies that more events of that type are needed to create a meaningful difference, suggesting each individual event has less impact. For life‐or‐death outcomes, clinicians and patients may act on very small absolute differences (e.g., < 1 per 1000), while for less severe outcomes (e.g., postoperative VTE), a larger difference (on the order of 10–15 per 1000) is typically needed before the benefit clearly outweighs the harms or inconvenience. Thus, the relationship between MIDs and relative values is inverse when expressing impact relative to death.
  For example, if the MID for death is 1/1000 and the MID for VTE is 3/1000, then the RV for VTE relative to death is 0.33. This transformation *enables standardized comparison* across different outcomes and helps quantify patient preferences in a way that can be incorporated into formal decision models. By expressing outcomes in terms of relative values, clinicians and researchers can more easily compare and communicate the importance of different health states.
3. Use the drop‐down menu to select the certainty of evidence (CoE).
  The GRADE SoF includes an assessment of CoE (high, moderate, low, or very low), reflecting our confidence in the accuracy of the estimated intervention effects [6]. Evidence appraised as “high” certainty is more likely to be close to the truth, whereas “low(er)” certainty evidence may reflect true, exaggerated, or underestimated effects [6]. Meta‐epidemiological studies show that high‐quality evidence without methodological deficiencies is trustworthy, while lower‐quality evidence may lead to biased estimates [17, 18, 19]. We previously reported that the probability of a “true” estimate decreases by about 20% for each level drop in CoE—from over 80% for high, to 55% for moderate, 35% for low, and 15% for very low CoE [17].

Thus, when a systematic review (SR) team reports high CoE, we assume the treatment effects fall within the original 95% confidence intervals reported in the SoF tables. For lower CoE, it is not possible to accurately predict the direction or size of the effect. Therefore, for lower certainty of evidence (CoE), we widen the confidence intervals to reflect the increased uncertainty.

For example, in Figure 1, the CoE for all outcomes was deemed high. However, if the CoE were moderate, low, or very low, we would allow the distribution of effects over 97%, 99%, and 99.9% confidence intervals, respectively. These uncertainties are then incorporated into the sensitivity analysis (see *Output* section). [Note that a user can override these default distributions as they see fit.]

If the goal is simply to determine the ΔEU—which we recommend as the default approach to assess which intervention is better—then no further steps are required. This entire exercise typically takes less than a few minutes.

To determine **differences in QALY and LE**, a few additional steps are required:

### 1.2 Ensure That the SoF Tables Report Events Per Unit of Time

To convert ΔEU into QALY and LE gains, the data in the SoF tables must be reported as annual event rates. For example, the evidence table copied from the original GRADE SoF and included in the Excel calculator reports both the total number of events per 1,000 people and their annualized risk (see the ‘original SoF table’ tab in the Excel file) [20]. If the SR team did not report annualized risks, the user will need to convert the reported event rates into annual event rates manually.

From the DEALE equations, it is clear that the mortality rate consists of **background (age‐ and sex‐related) mortality** plus **disease‐specific mortality**, together comprising the **overall (compound) annual mortality** [8, 9]. The background mortality can be obtained from life tables, such as the U.S. Social Security tables, which are built into our calculator. Disease‐specific mortality is obtained from the study reports. This means that the overall mortality reported in a study reflects the background mortality of the patients' average age included in that study. Consequently, the disease‐specific mortality must be **lower than** the compound (overall) mortality.

For example, in a study on the duration of secondary treatment to prevent recurrent venous thromboembolism [20], the investigators reported an annual overall (compound) mortality rate of 7 per 1000. However, the mean age of participants ranged from 41 to 70.1 years, which according to DEALE method corresponds to the annual background mortality rates of 0.027996 to 0.073046 for males, and 0.024907 to 0.0625 for females, respectively. This clearly suggests that the investigators were reporting VTE‐specific mortality rather than overall mortality.

To indicate this in the Excel calculator, first select **“1”** from the drop‐down menu to denote that the outcome represents mortality. *(Note: If there is more than one mortality outcome, assign ‘1’ to only one of them to avoid double counting.)*

Next, under **“age of study population”**—listed as the last entry among all possible age values—select **“Dis. Spec”** to indicate that the study reported only **disease‐specific mortality** for the given patient population, and not the overall (compound) mortality.

*(Correct selection of this option will result in the output in cell D11 displaying ‘1E‐05.’)*

Then, use the drop‐down menus to select the **age** and **gender** of the patient for whom you are interested in calculating QALY and LE gains.

If the evidence table includes **true overall (compound) mortality**, simply select the **mean** or **median** age of the study population. If you are only interested in calculating QALY/LE for the **study population**, select the **same age and gender** under both “age of your patient” and “gender of the patient.” However, if you wish to **individualize the results** for another population of interest, select the appropriate age and gender as needed.

## 2 Results

Based on the given Inputs in the calculator, we can see **Outputs** presented as (Figure 1b):

1. Net Benefits [ΔEU] with Associated Uncertainty Interval and Monte Carlo Sensitivity Analysis.
  The treatment with higher (positive) values of ΔEU yields greater net benefits and is therefore considered the better treatment option. The output includes both the uncertainty interval, and a sensitivity analysis performed via Monte Carlo simulation.
  Monte Carlo simulation effectively transforms a deterministic ΔEU model into a probabilistic one. It translates uncertain evidence into a distribution of possible outcomes, enabling the assessment of both the expected value of competing strategies and the credibility of the recommendations under the evidentiary uncertainty captured in the SoF table.
  By default, the calculator performs 10,000 iterations, drawing random values for each uncertain model parameter from pre‐specified normal probability distributions as defined in the SoF table. These default values can be modified if needed.
  In the example included in our calculator, short‐term anticoagulation was found to be inferior to long‐term anticoagulation, with ΔEU = EU[ST_AC] − EU[LT_AC] = –0.019 (95% uncertainty interval: –0.027 to –0.011). The Monte Carlo simulation shows that the overall distribution of plausible ΔEU values remained negative, consistently favoring long‐term anticoagulation.
  As a general rule, a strategy that is favored in *more than 90% of iterations* is considered highly **robust**.
2. QALY and Life Expectancy Gains.
  Quality‐adjusted life years (QALYs) assess the value of medical interventions by combining both the **quantity** and **quality** of life they provide. We used relative values (RV) and minimal important differences (MID) to assess quality‐of‐life utility values. Unlike standard decision analyses—where health states are assigned values between 0 and 1 (with 0 representing death and 1 representing perfect health) [7]—in our model, we anchor death at 1 and compare other health utility values relative to this anchor (as described above).

One QALY equates to 1 year of life in perfect health. If a person's health is less than perfect, the QALY is adjusted proportionally. QALYs can be contrasted with life expectancy (LE) to assess how many years lived in perfect health are equivalent to years lived in imperfect health. For example, living 2 years with a health utility of 0.5 results in 2 years × 0.5 = 1.0 QALY, which is equivalent to 1 year in perfect health.

In our example, for a 60‐year‐old man, we calculated:

- **QALY** = **12.63** for short‐term anticoagulation
- **QALY** = **16.6** for long‐term anticoagulation

Thus, long‐term anticoagulation yields approximately **4 additional QALYs** compared to short‐term treatment. Similarly, long‐term treatment resulted in about **1 additional year of life expectancy** (LE: 18.8 vs 17.8 years). Monte Carlo sensitivity analysis also confirmed the superiority of long‐term anticoagulation across a wide range of simulated scenarios.

## 3 Discussion

Let us provide some additional useful information on how to interpret QALY and LE gains.

In general, *QALY gains* exceeding 0*.32–0.5 (equivalent to 4–6 months of perfect health)* are considered **large** treatment effects [21]. *LE gains* of *1 month for average‐risk populations or 1 year for high‐risk populations* are also considered **large** effects [22].

Given these **benchmarks**, one might question whether our estimates overstate the benefits of long‐term anticoagulation. This is unlikely, as the underlying evidence on the duration of anticoagulation is considered to be of high quality. However, disease‐specific mortality was unusually low, which—as explained below—may have led to overestimated effects.

We should remain mindful of the **limitations of the DEALE model** [8, 9].

- The DEALE method is most accurate when **disease‐specific mortality exceeds 10% per year**, which is common in fields such as oncology.
- For **patients older than 50 years**, DEALE provides an excellent approximation regardless of disease‐specific mortality.
- For **patients younger than 50 years**, accuracy depends on the disease‐specific mortality rate:

**Table:** 

Disease‐specific mortality rate	Age	Error in LE
> 10%/year	Any	< 1 year
5–10%/year	40	~1.5 years underestimation
< 5%/year	40	Up to 4 years underestimation

The error increases in younger patients with lower disease‐specific mortality rates.

Importantly, the calculation of ΔEU is not affected by the limitations of the DEALE method. When ΔEU is consistent with the QALY and LE estimates, the alignment across all three metrics further supports the credibility of our results.

## 4 Conclusion

By converting expected utility metrics into more intuitive measures like QALY and life expectancy, we believe we are one step closer to achieving **full integration of EBM with decision‐analytical frameworks**—the goal we set for the next quarter‐century of progress in EBM [6].

## Conflicts of Interest

The authors declare no conflicts of interest.

## Supporting information

DeltaEU LE and QALY ‐JECP.

## Data Availability

All data and the EXCEL‐based software application are provided in the Supplementary Material.
